# Identification of novel natural compound inhibitors for human complement component 5a receptor by homology modeling and virtual screening

**DOI:** 10.1007/s00044-016-1591-1

**Published:** 2016-05-05

**Authors:** Faraz Shaikh, Shirley W. I. Siu

**Affiliations:** E11-4025, Faculty of Science and Technology (FST), University of Macau, Avenida da Universidade, Taipa, Macau, China

**Keywords:** C5aR, Natural compound inhibitor, Homology modeling, Virtual screening, Molecular docking, MM-GBSA

## Abstract

**Abstract:**

Neuropathic pain and inflammatory pain are two common types of pathological pain in human health problems. To date, normal painkillers are only partially effective in treating such pain, leading to a tremendous demand to develop new chemical entities to combat pain and inflammation. A promising pharmacological treatment is to control signal transduction via the inflammatory mediator-coupled receptor protein C5aR by finding antagonists to inhibit C5aR activation. Here, we report the first computational study on the identification of non-peptide natural compound inhibitors for C5aR by homology modeling and virtual screening. Our study revealed a novel natural compound inhibitor Acteoside with better docking scores than all four existing non-peptidic natural compounds. The MM-GBSA binding free energy calculations confirmed that Acteoside has a decrease of ~39 kcal/mol in the free energy of binding compared to the strongest binding reference compound. Main contributions to the higher affinity of Acteoside to C5aR are the exceptionally strong lipophilic interaction, enhanced electrostatics and hydrogen bond interactions. Detailed analysis on the physiochemical properties of Acteoside suggests further directions in lead optimization. Taken together, our study proposes that Acteoside is a potential lead molecule targeting the C5aR allosteric site and provides helpful information for further experimental studies.

**Graphical Abstract:**

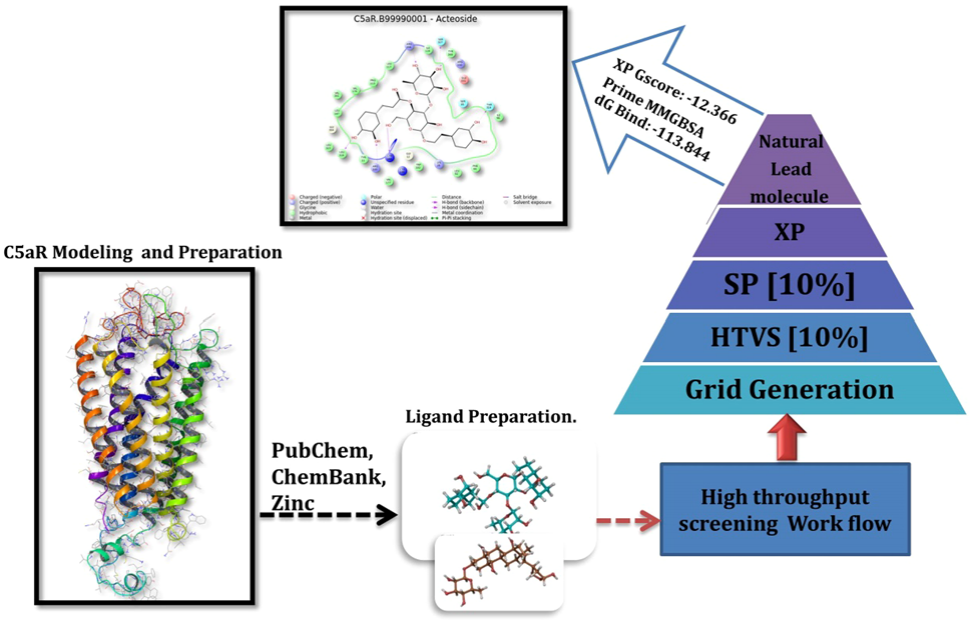

## Introduction

Natural products derived from plants, fungi, bacteria, and marine organisms, which have long been utilized in Western and Eastern medicine, are widely investigated by modern science as ideal candidates for drug therapy. These natural products are usually secondary metabolites that can be easily metabolized by the human body and are promising alternatives to synthetic drugs (Butler, 2004). They have been found to apply in a plethora of pharmaceutical applications, which includes the use in treating inflammatory, parasitic, metabolic, oncological, and pain-related conditions (Saklani and Kutty, 2008). This emerging trend in pharmaceutical research is fueled by the advances in ancillary technologies including spectroscopic, chromatographic, biosynthetic, and synthetic methods, as well as the ease in obtaining these isolates by synthetic or combinatorial chemistry and molecular modeling (Ley and Baxendale, 2002).

The most common types of pathological pain include inflammatory and neuropathic pain both of which represent important health problems. While inflammatory pain is a crucial and classic manifestation of the inflammatory process, neuropathic pain can arise from any of multiple nerve lesions or diseases, with symptoms including hyperalgesia or allodynia (Kidd, 2001; Baron *et al*., 2010). Even painkillers, like opioids and nonsteroidal anti-inflammatory drugs (NSAID) which are generally very efficient, are only partially effective; even worse, studies revealed that prolonged exposure to these painkillers can cause adverse effects (Devulder *et al.*, 2009). Consequently, there is tremendous demand for identifying or developing new chemical entities to combat pain by exploiting alternative biological mechanisms that will involve fewer side effects.

Based on recent results from several animal models for neuropathic pain, the G protein-coupled receptor C5aR has emerged as one of the potential pharmacological targets for treating neuropathic pain (Moriconi *et al*., 2014). As it is widely expressed in inflammatory cells, C5aR binds to anaphylatoxin C5a generated during complement system activation to result in an effective clearance of infectious agents (Gerard and Gerard, 1991). However, under various pathological conditions, overproduction of C5a can down regulate immune response and at the same time over activate other cell types. This can lead to uncontrolled inflammation and trigger proinflammatory and immunosuppressive disorders (Guo and Ward, 2005). Therefore, finding antagonists to inhibit C5aR signaling provides a promising way for improving the treatment of chronic pain.

Despite significant efforts over the past decades, the development of peptide as antagonist regulator for C5aR has been hampered by several issues related to cost effectiveness and low oral bioavailability, lack of potency, low selectivity, and poor drug-like properties. The most successful so far has been the PMX-53, a cyclic peptidomimetic antagonist designed to mimic the C-terminal portion of C5a (Finch *et al*., 1999). Though it has shown encouraging results in preclinical studies, its development remains limited by its short half-life and disapproving bioavailability (Ricklin and Lambris, 2007).

Previous studies have indicated that a minor pocket spanning between transmembrane (TM) 1, 2, 3, 6, and 7 is a key motif for the intracellular activation process. A study by Alessio Moriconi and coworkers successfully combined the information on structural and functional features of allosteric sites in homologous chemokine receptors, to perform de novo design of a new class of allosteric small molecular weighted inhibitors of C5aR (Hodes *et al*., 2014).

As mentioned earlier, natural ligands have certain advantages over synthetic ones in terms of minimal side effects. To cope with the increasing demand for non-peptidic antagonists with high specificity against C5aR, in this work, we performed computational studies of C5aR and attempted to identify non-peptidic natural compounds as potent inhibitors. To this end, we generated the three-dimensional structure model of C5aR using comparative homology modeling and subsequently screening a library of approximately 1500 natural compounds toward the TM regions at the allosteric site of C5aR.

## Materials and methods

### Homology modeling of the structure of C5aR

A comparative modeling approach was implemented for modeling of C5aR protein of the GPCR family. The 350 amino acid long protein sequence of C5aR (Accession no: P21730) was retrieved from UniProt database in FASTA format. PDB structures sharing a high degree of homology with C5aR protein were identified as templates (PDB IDs: 2LNL, 2LOT, 4EA3, and 3PBL) on the basis of parameters such as lowest *E* values, highest score, and the most aligned regions by position-specific iterated basic alignment search tool (PSI-BLAST) and global alignment with the query sequence. These templates were used to generate homology models of C5aR using the multiple template modeling approach using MODELLER 9.14 (Sali and Blundell, 1993). Furthermore, this model structure was subjected to assess with the DOPE score (Shen and Sali, 2006) and Ramachandran plot (Ramachandran *et al*., 1963). It is remarked that since C5aR is a transmembrane protein, most validation tools such as Profile 3D and QMEAN (Benkert *et al*., 2011), which are statistical methods derived from soluble protein structures, are not suitable for assessment.

### Protein model preparation

Using the *Preparation wizard* of Schrödinger software, the C5aR homology model was processed through the steps of water removal, bond order assignment, and addition of hydrogen atom. It was then energy minimized using default constraints of 0.30 Å RMSD using the OPLS-2005 force field.

Since C5aR contains helix-connecting loops which are involved in the ligand binding site, the Prime module in Schrödinger was invoked for loop refinement. Prime loop prediction is an ab initio method, and it generates structures of the loop segment by reference to a backbone dihedral library. The generated loop structures are clustered, scored, side chain refined, and energy minimized; only the best scored structure is returned. While there is no perfect loop modeling method at the moment, a recent assessment of loop prediction methods revealed that only Prime is able to generate loop structure with <2.5 Å for loops up to 10 residues, while other methods (such as ICM, Sybyl, and MODELLER) up to 7 residues (Rossi *et al.,*^2007^).

To assure the quality of the refined homology model, MD simulation was conducted with the protein embedded in POPC lipid bilayer and solvated in explicit water using GROMOS 53A6 force field (Oostenbrink *et al.,*^2004^) for protein and GROMOS 53A6L lipid force field for the membrane (Poger and Mark, 2012). The protein RMSD gave an average of ~5.7 Å in 10 ns, indicating that there were moderate fluctuations of the structure; we noted that these fluctuations were focused in the intracellular region (specifically, residue 243 to 266, root-mean-square fluctuations (RMSF) >5 Å) which was far away from the ligand binding site. Our analysis also showed that binding site residues had very low RMSF values of <2 Å. These results proved the validity of our homology model for undertaking further docking studies.

### Ligand library preparation

The ligand library was compiled from various databases and from literature survey. A total of 1500 natural small molecules were retrieved in SDF format from PubChem database (Bolton *et al*., 2008) and ZINC (Irwin *et al*., 2012). These small natural compounds were prepared using the *ligprep* module of Schrödinger by assigning the bond orders and angles. Furthermore, those molecules were subjected to minimization using the OPLS-2005 force field.

### Grid generation

The C5aR structure was subjected to SiteMap analysis (Halgren, 2009) and yielded five active sites. Based on the SiteScore values, site 1 was chosen to perform molecular docking studies. The active sites predicted by SiteMap are Gln 149, Ala 193, Asp 255, Leu 264, Ile 223, and Glu 191. The grid box was generated around the minor pocket spanning between TM-1, -2, -3, -6, and -7. This region was set as the centroid using the *Receptor grid generation* tab of the Glide module in Schrödinger.

### QikProp analysis

The QikProp module (Qikpro 4.2 2014) of Schrödinger was used for efficient evaluation of pharmaceutically relevant properties of natural compounds library; it predicts the Absorption, Distribution, Metabolism, Elimination (ADME) properties of all natural compounds. The compounds which were screened by Glide and their predicted ADME properties are discussed in the next section.

### Virtual screening

High throughput virtual screening was implemented by Schrödinger software through the virtual screening workflow of Glide. Three steps were executed according to the workflow, which includes HTVS, SP (standard-precision) docking, and XP (extra precision) docking. Based on this screening process, we have screened the 1500 natural compound library against the C5aR structure. Compounds which were screened successfully from HTVS were further subjected to SP docking for higher precision docking to get more accurate results. Furthermore, XP docking was carried out to remove the false-positive results.

### Binding free energy calculation

Next to docking, Prime Molecular Mechanics/Generalized-Born/Surface Area (MM-GBSA) (Prime 2.1, 2009) (Rastelli *et al.*, 2010) was used for the calculation of binding free energy for the docked complexes. The method used the following equation:

1 $$\Delta {\text{Gbind }} = \, \Delta {\text{EMM }} + \, \Delta {\text{GSOLV }} + \, \Delta {\text{GSA}},$$

where ΔEMM is the difference in the minimized energies between the C5aR–inhibitor complex and the total of energies of unliganded C5aR and inhibitor; ΔGSOLV is the difference in the GBSA solvation energy of the protein–inhibitor complex and the total of the solvation energies for the unliganded C5aR and inhibitor; ΔGSA is the change in surface area energies for the complex and the totality of the surface area energies for the unliganded C5aR and inhibitor. All ligand poses were further minimized using the local optimization by Prime where the energies of complexes were calculated with the OPLS-2005 force field and Generalized-Born/Surface area continuum solvent model. While the simulation process, the ligand strain energy was also taken into consideration.

## Results and discussion

### The C5aR homology model

**Fig. 1:**
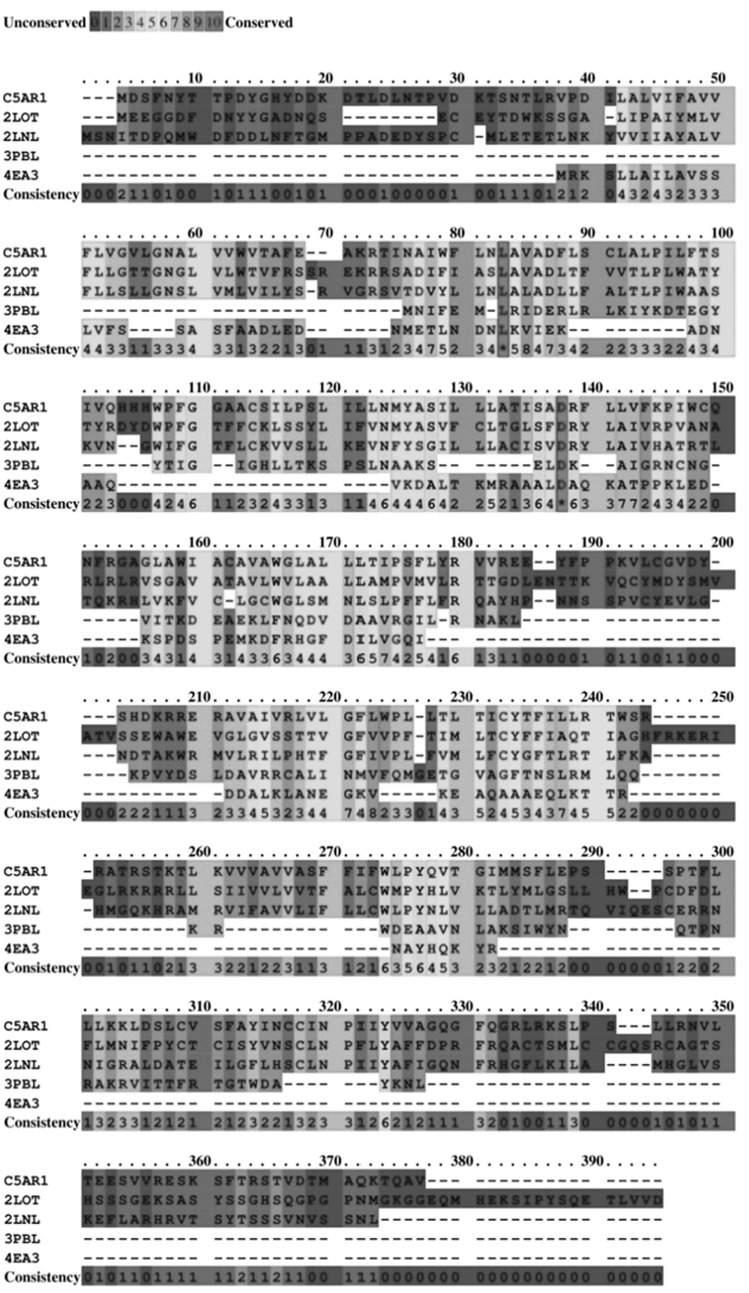
ClustalW2 result showing the sequence alignment of the template proteins (PDB IDs: 2LOT, 2LNL, 3PBL and 4EA3) with that of the human C5aR sequence

**Fig. 2:**
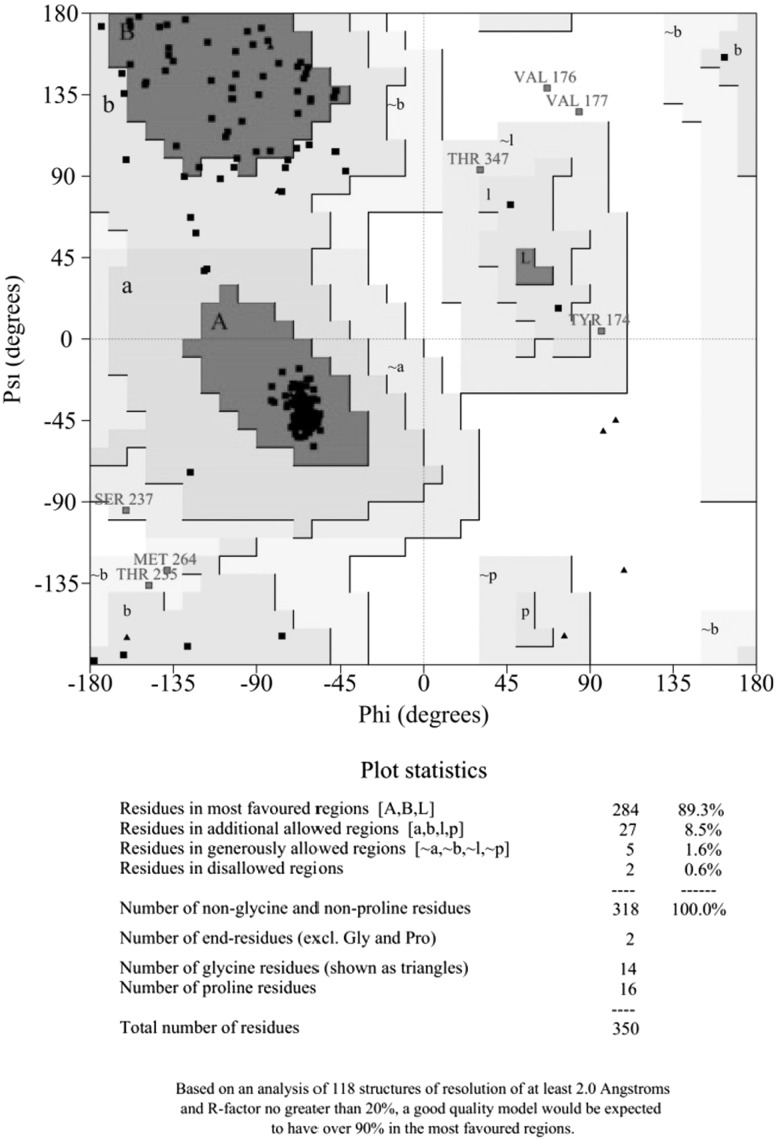
Ramachandran plot of the C5aR homology model. *Color red* indicates low-energy regions, *yellow* allowed regions, *pale* *yellow* the generously allowed regions, and *white* disallowed regions (Color figure online)

Owing to unavailability of experimental structure of the C5aR protein, homology modeling was employed to obtain the tertiary structure. This was accomplished by taking crystal structure of other GPCR proteins which shared a high degree of sequence similarity and lowest E values with C5aR. Figure 1 shows the ClustalW2 multiple sequence alignment result of the template PDBs—2LNL, 2LOT, 4EA3, and 3PBL, against the query sequence C5AR1_HUMAN. All template proteins showed an identity of ~40 % each and with a query coverage of >90 % in the amino acid region 160–240 and 270–280. Multiple sequence alignment shows a high degree of conservation of the amino acid residues involved in non-peptide binding site in all four templates used for modeling. Using the MODELLER program, five models were generated taking the multiple templates. The model with the lowest DOPE score was subjected to further structural assessment. As shown in Fig. 2, the Ramachandran plot of the C5aR model calculated that 97.8 % of all amino acids resided in the most favored and additionally allowed regions. These data suggest that the C5aR homology model was of good quality and did not suffer any serious steric clashes.

### Library of natural compounds and ADME-based filtering

A comprehensive search for all natural compounds with either characterized anti-inflammatory property or those compounds with proven efficacy against Neuropathic pain was made by searching the electronic literature on the PubChem and ZINC database. All studies published between 1970 and June 2015 in English language was included in the analysis. A total of 1500 compounds were identified which fulfilled the aforementioned criterion.

The enlisted natural compounds were filtered based on their ADME properties using QikProp. The compounds prepared were subjected to the druglikeness filter. The criteria of the filter were set as follows: molecular weight within 160–480, number of heavy atoms within 20–70, lipophilicity within 40–130, number of hydrogen bond donors within 4–7, number of hydrogen bond acceptors within 8–12. All the ligands constituting the library of natural compounds conformed to the above-mentioned criterion and were subjected to the docking analysis using Glide.

### Identification of binding site

**Fig. 3:**
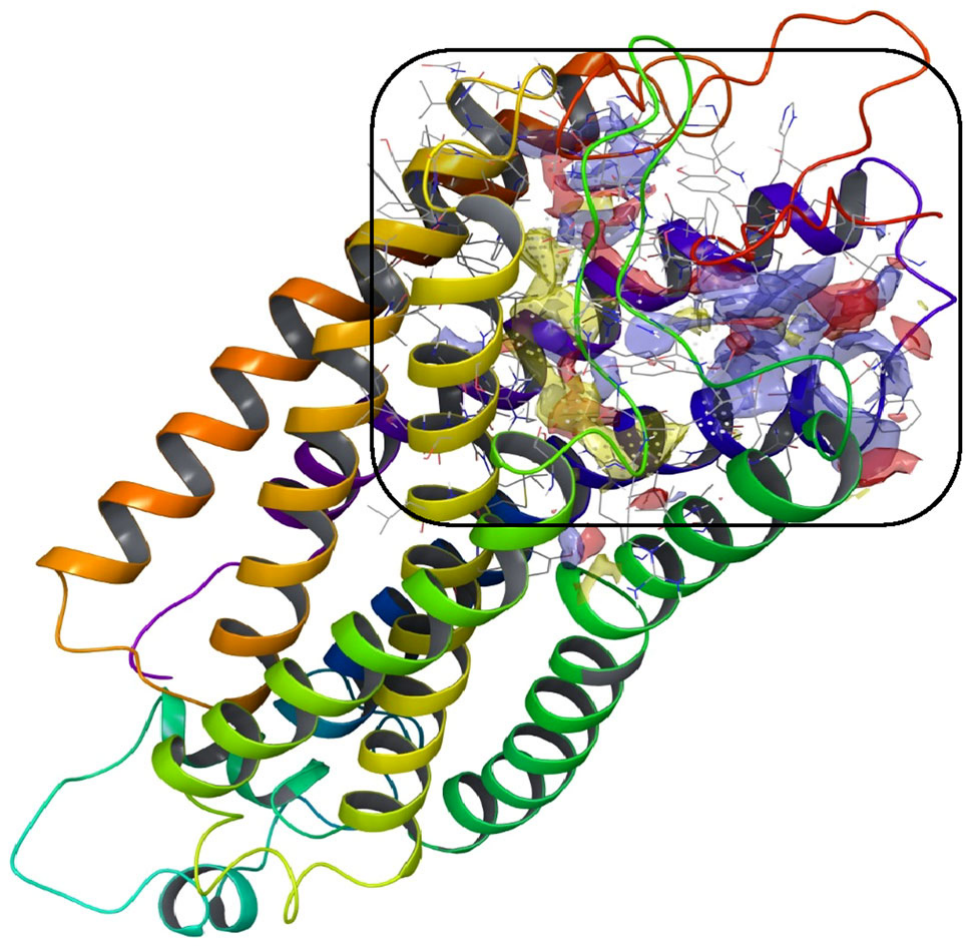
Image displays SiteMap-1 identified in the SiteMap analysis using the C5aR structure. The *rectangle* indicates the location of the generated grid box used in further molecular docking studies. The *different color* meshes show regions that have hydrophobic residues (*yellow*), hydrogen bond donors (*blue*), and hydrogen bond acceptors (*red*) (Color figure online)

In accordance with previously available report, C5aR structure is known to consist of three binding sites (Bowie *et al.*, 1991) and choosing the right one as the target for the antagonist is a non-trivial task. To identify the correct binding site, SiteMap analysis was performed on the C5aR structure after the protein preparation steps. In total, SiteMap recognized five active sites. By considering the SiteScore and previously reported literature available about the C5aR binding sites (Halgren, 2007), SiteMap-1 was chosen for further molecular docking studies (Fig. 3). The predicted active site consists of residues T29, F182, P184, D191, P270, S271, S272, F275, S272, F275, and L276, where majority of them were previously reported in (Rana and Sahoo, 2015).

### Docking of reference compounds

**Fig. 4:**
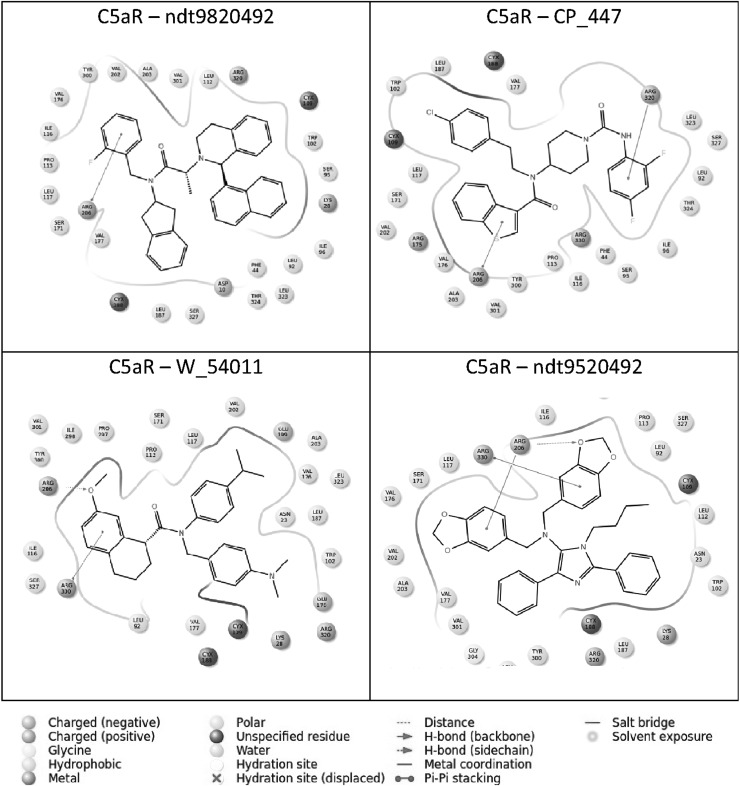
Ligand interaction map of NDT982042, CP_447, W_54011, and NDT9513727. The proposed interaction modes of all four reference compounds have been shown in the stick format. Residues involve in hydrogen bonding are represented as *pink dotted lines,* whereas pi–pi stacking are shown in *green color arrow lines* (Color figure online)

To set a cutoff value for docking studies, we docked all previously reported non-peptidic antagonist molecules including NDT9520492 (Waters *et al*., 2005), CP_447 (Blagg *et al*., 2008), NDT9513727 (Sumichika *et al*., 2002), and W_54011 (Ames *et al*., 2001) using the XP docking method. The results showed that NDT9520492 has the highest binding affinity toward C5aR with the XP GScore of −8.291 kcal/mol while CP_447, NDT9513727, and W_54011 have −7.281, −5.966, and −5.466 kcal/mol, respectively. Hence, for screening better lead structure, a cutoff GScore value of −6.00 kcal/mol was set as the filtering criteria in virtual screening (Fig. 4).

### Virtual screening of natural ligands

**Fig. 5:**
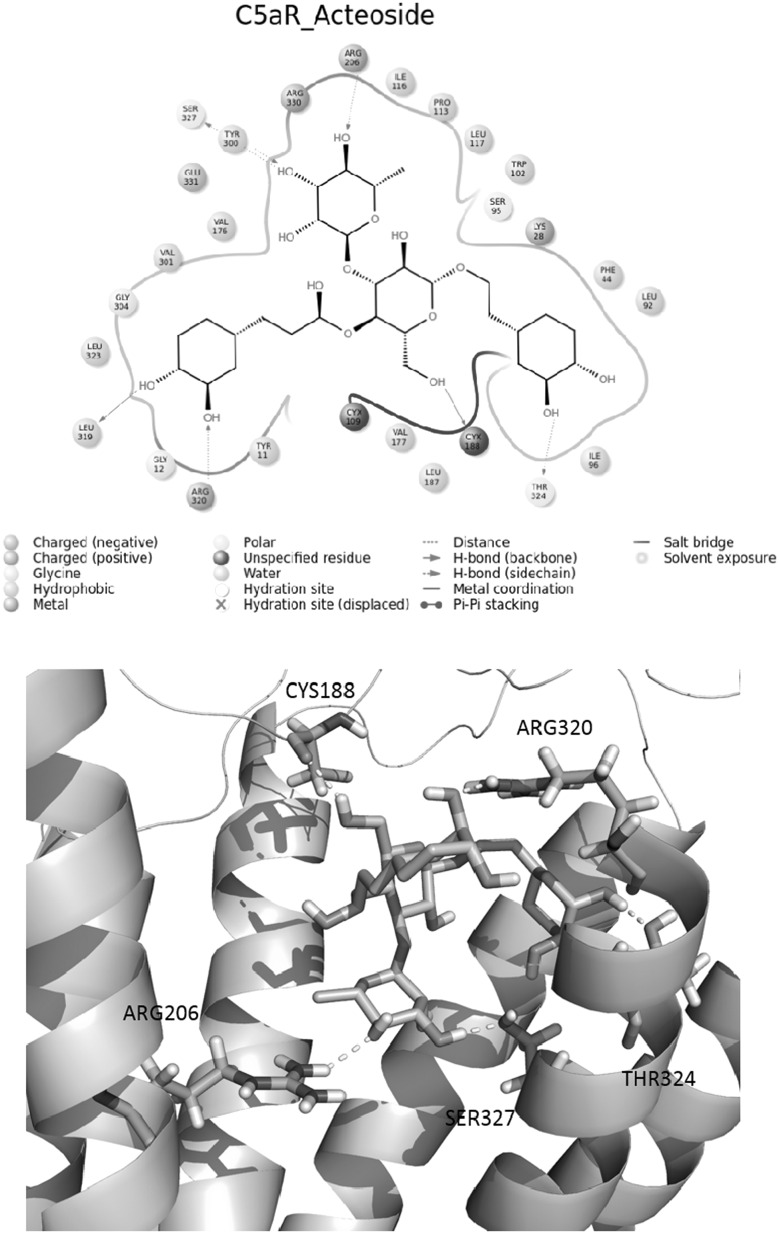
(*Top*) Ligand interaction map of the lead molecule Acteoside and (*bottom*) its proposed binding mode in the active site. The ligand (*orange*) and the interacting protein residues (*green*) are displayed in stick format. For better clarity, interacting residues TYR300, LEU319, the nonpolar hydrogens in the ligand and TM helix in the residue ID range 278–319 are not shown (Color figure online)

As shown in Table 1, results of virtual screening found two potential natural molecules with better GScore than the reference compounds. Remarkably, Acteoside (PubChem ID 5281800) with XP GScore of −12.366 kcal/mol showed highest binding energy than all four previously reported C5aR inhibitors. The ligand interaction map was generated to get insight into their interaction patterns. As shown in Fig. 5, the docked pose analysis of Acteoside revealed that it formed 7 hydrogen bonds with the binding site of C5aR, interacting with the pocket residues Arg206, Ser327, Tyr300, Leu319, Arg320, Cys188, and Thr324. There are several new residues reported here together with the crucial binding residue Arg206, which was found as interacting residue in all four reference compound complexes. The residue Arg206 is positioned near the cytoplasmic surface; it is helpful in recognition of allosteric pocket; and several cyclic peptide antagonists are known to interact with Arg206 (Kaneko *et al*., 1995). It is evident that reference compounds NDT9520492, CP_447, NDT9513727, and W_54011 are also involved in hydrogen bonding and pi–pi interaction with the key residues Arg206 and Arg330.

**Table 1:** GScores and the energy components of the top-ranked natural compounds and the reference compounds

Ligand	XP GScore	Glide Evdw	Glide Ecoul	Glide Einternal	XP HBond	G5aR residues with hydrogen bond with docked ligand
Acteoside	−12.366	−35.926	−22.838	13.976	−5.283	Arg206, Ser327, Tyr300, Leu319, Arg320, Cys188, Thr324
Toxicarioside	−8.871	−48.593	−10.579	5.321	−1.92	Tyr300, Ala303
NDT9520492	−8.291	−45.214	−14.721	7.249	−1.86	Arg206
CP_447	−7.281	−39.141	−19.084	4.341	−2.52	Arg206, Arg320
NDT9513727	−5.966	−23.561	−9.279	5.178	−0.98	Arg206, Arg330
W_54011	−5.466	−31.145	−8.324	4.214	−1.74	Arg206, Arg330

All energy values are in kcal/mol. C5aR protein residues having hydrogen bonds with the docked ligands are also listed in the last column

### Prime MM-GBSA calculation

The top-ranked molecules were further subjected to Prime MM-GBSA calculations. Molecular Mechanics/Generalized-Born/Surface Area (MM/GBSA)-based relative binding free energy quantification has emerged as an effective tool to understand mutational effect in large biomolecular systems (Massova, 2000). As presented in Table 2, the two novel natural ligands Acteoside and Toxicarioside were found to have the strongest binding free energy (dG_bind) of −113.884 and −90.083 kcal/mol, respectively, whereas reference compounds have weaker binding free energies in the range of −43 to −75 kcal/mol. Main contributions to the tight binding of Acteoside to C5aR are the exceptionally strong lipophilic interaction (dG_bind_Lipo), enhanced electrostatics (dG_bind_Coulomb) and hydrogen bond interactions (dG_bind_Hbond). Toxicarioside does have strong hydrophobic interaction with the receptor protein, but it has largely reduced electrostatic interaction making it a less tight-binding ligand.

**Table 2:** Binding free energies and the energy components of the C5aR–ligand complexes from MM-GBSA calculations

Ligand	dG_bind	dG_bind_Coulomb	dG_bind_Lipo	dG_bind_Covalent	dG_bind_Hbond
Acteoside	−113.884	−41.613	−78.574	9.886	−2.993
Toxicarioside	−90.083	−3.773	−67.48	3.56	−0.491
NDT9520492	−75.062	−39.54	−34.25	7.23	−0.172
CP_447	−63.251	−36.23	−29.47	4.92	−0.103
NDT9513727	−45.442	−17.15	−26.12	3.09	−0.050
W_54011	−43.251	−21.31	−23.57	5.04	−0.190

All energy values are in kcal/mol

### Comparison of physiochemical properties of Acteoside and reference compounds

Considering Acteoside as a more potent inhibitor, we performed QikProp analysis on it and compare to the results of reference compounds. QikProp is a quick and ADME program to predict physiochemical descriptors and pharmaceutically relevant properties of organic molecules. For each descriptor, the range satisfying 95 % of known drugs is also provided for comparison.

As shown in Table 3, Acteoside is a molecule with the largest molecular weight and possesses the largest solvent accessible surface area and more hydrophobic components compared to reference compounds. It possesses exceptionally large number of hydrogen bond donors and acceptors. Among the 6 predicted partition coefficients, 4 of them fall within the recommended ranges: water/gas (QPlogPw), aqueous solubility (QPlogS), brain/blood (QPlogBB), and skin permeability (QPlogKp), whereas octanol/gas partition coefficient (QPlogPoct) is beyond the recommended range and octanol/water partition coefficient (QPlogPo/w) is below.

**Table 3:** QikProp results of the top-ranked natural ligand Acteoside and four reference compounds

Ligand	mol MW	SASA	FOSA	donorHB	accptHB	HOA	QPlogPoct	QPlogPw	QPlogPo/w	QPlogS	QPlogBB	QPlogKp
Reference range	130–725	500–2000	0–750	0.0–6.0	2.0–20.0	1: low, 2: medium, 3: high	8.0–35.0	4.0–45.0	−2.0–6.5	−6.5–0.5	−3.0–1.2	−8.0–1.0
Acteoside	640.721	940.26	549.74	10	25.5	1	46.93	39.548	−3.011	−1.658	−2.125	−6.605
NDT9520492	554.706	804.246	175.264	0	5	1	24.171	10.658	6.978	−6.458	0.578	−1.415
CP_447	554.053	833.107	135.432	1	5	1	24.46	11.839	7.203	−8.761	−0.105	−0.774
NDT9513727	559.663	785.734	241.02	0	5	1	23.171	8.476	7.99	−7.186	−0.026	0.911
W_54011	456.627	813.867	522.592	0	4.75	1	20.274	6.794	7.246	−8.013	−0.122	−0.373

The QikProp descriptors are (columns from left to right): molecular weight (mol MW), total solvent accessible surface area (SASA), hydrophobic component of the SASA (FOSA), estimated number of hydrogen bond donor (donorHB) acceptor (accptHB) in aqueous solution, and Human Oral Adsorption (HOA). QPlogPoct, QPlogPw, QPlogPo/w, QPlogS, QPlogBB, and QPlogKp are predicted partition coefficients of octanol/gas, water/gas, octanol/water, aqueous solubility, brain/blood, whereas QPlogKp is predicted skin permeability. Numbers in brackets are range for 95 % of known drugs or recommended values

It is noted that although all ligands are classified as low in human oral absorption (HOA) Acteoside has the highest predicted aqueous solubility of −1.658 (QPlogS) compared to those in the reference compounds (−8.761 to −6.458, 3/4 of them are out of the recommended range of known drugs). As low solubility limits absorption and causes low oral bioavailability, a high oral bioavailability drug potentially reduces the amount of administered drug necessary to achieve the desired pharmacological effect while reducing the risk of side effects and toxicity. Based on these prediction results, it is suggested that Acteoside may be a more potent drug candidate regarding its efficacy than reference compounds.

Nevertheless, the three partition values octanol/gas, water/gas, and octanol/water together suggest that Acteoside has low lipophilicity which may lead to low gastrointestinal absorption by the way of passive diffusion. For diseases of the brain, another important consideration of drug candidate is its brain penetration property. Although within the recommended range, the predicted brain/blood partition coefficient (QPlogBB) of Acteoside is only −2.125, the smallest among all ligands. Both undesirable properties of Acteoside can be explained by the large number of hydrogen bond donors and acceptors in its structure. While hydrogen bonds can only contribute about −3 kcal/mol to the free energy of binding (see Table 2), further optimization of the Acteoside structure may consider eliminating selected hydrogen bond donors or acceptors in order to enhance its lipophilicity and thus its blood–brain barrier permeability for drug targeting neuropathic pain diseases.

## Conclusion

In recent times, there has been an increased demand for identifying or developing new chemical entities to combat neuropathic and inflammatory diseases by exploiting alternative biological mechanisms that will involve fewer side effects. As a critical first step to drug design for neuropathic and inflammatory diseases, a computational study of the human C5aR was performed with the goal to identify lead compounds from natural products. Using an in-house prepared library of 1500 natural compounds, and by virtue of homology modeling and virtual screening, two novel natural compounds with low binding free energies than reference compounds were identified. Energy calculations from docking, MM-GBSA, and ADME analysis with QikProp unambiguously reveal that Acteoside is a more potent lead compound with stronger binding interactions with C5aR than currently known compounds.

Indeed, looking back into the literatures, the anti-inflammatory role of Acteoside has been confirmed previously by many experimental groups (Lee *et al*., 2006; Seo *et al*., 2013). The compound has been used in Ayurvedic and Chinese medicine for many years as an anti-inflammatory agent and has a large range of pharmacological and biochemical effects (He *et al.*, 2011) including anti-tumor (Inoue *et al.*, 1998), anti-nephritic (Hayashi *et al*., 1994), anti-hepatotoxic (Lee *et al*., 2004), and antiseptic (Lee *et al*., 2005) activities. Acteoside was also found to inhibit the high-mobility group box 1 (HMGB1) release in vitro and decrease serum and lung HMGB1 levels in CLP-induced sepsis in vivo (Lee *et al*., 2006), presumably related to the binding of C5a to the C5L2 protein (Rui *et al*., 2012), a closest homology with C5aR. While the experimental structure of neither C5aR nor C5L2 is available, result from pairwise sequence alignment of the human C5aR and C5L2 (result not shown) reveals that the major interacting residues at the active site ARG206, TYR300, CYS188 are conserved in them, suggesting that Acteoside may bind in them with a similar binding mode. Nevertheless, this is still to be verified by further computational study via careful homology modeling and molecular docking.

As a conclusion, our computational study proposes Acteoside to be a better lead compound than all known inhibitors. This promises a new gateway for the further development of Acteoside as a potential anti-inflammatory agent targeting the C5aR allosteric site. Biological experiments to validate this inhibitor are being planned as a future work.
